# Neuroimaging and Cerebrovascular Changes in Fetuses with Complex Congenital Heart Disease

**DOI:** 10.3390/jcm11226740

**Published:** 2022-11-14

**Authors:** Flaminia Vena, Lucia Manganaro, Valentina D’Ambrosio, Luisa Masciullo, Flavia Ventriglia, Giada Ercolani, Camilla Bertolini, Carlo Catalano, Daniele Di Mascio, Elena D’Alberti, Fabrizio Signore, Antonio Pizzuti, Antonella Giancotti

**Affiliations:** 1 Department of Maternal and Child Health and Urological Sciences, Umberto I Hospital, Sapienza University of Rome, Viale del Policlinico 155, 00161 Rome, Italy; 2 Department of Experimental Medicine, Umberto I Hospital, Sapienza University of Rome, Viale del Policlinico 155, 00161 Rome, Italy; 3 Department of Radiological, Oncological and Pathological Sciences, Policlinico Umberto I, Sapienza University of Rome, Viale del Policlinico 155, 00161 Rome, Italy; 4 Pediatric and Neonatology Unit, Maternal and Child Department, Sapienza University of Rome (Polo Pontino), 4100 Latina, Italy; 5 Department of Radiology and Imaging Sciences, Santo Spirito Hospital, Lungotevere in Sassia 1, 00193 Rome, Italy; 6 Obsetrics and Gynecology Department, USL Roma2, Sant’Eugenio Hospital, 00144 Rome, Italy

**Keywords:** congenital heart disease, brain abnormalities, Doppler velocimetry

## Abstract

**Background:** Congenital heart diseases (CHDs) are often associated with significant neurocognitive impairment and neurological delay. This study aims to elucidate the correlation between type of CHD and Doppler velocimetry and to investigate the possible presence of fetal brain abnormalities identified by magnetic resonance imaging (MRI). **Methods:** From July 2010 to July 2020, we carried out a cross-sectional study of 63 singleton pregnancies with a diagnosis of different types of complex CHD: LSOL (left-sided obstructive lesions; RSOL (right-sided obstructive lesions) and MTC (mixed type of CHD). All patients underwent fetal echocardiography, ultrasound evaluation, a magnetic resonance of the fetal brain, and genetic counseling. **Results:** The analysis of 63 fetuses shows statistically significant results in Doppler velocimetry among the different CHD groups. The RSOL group leads to higher umbilical artery (UA-PI) pressure indexes values, whereas the LSOL group correlates with significantly lower values of the middle cerebral artery (MCA-PI) compared to the other subgroups (*p* = 0.036), whereas the RSOL group shows a tendency to higher pulsatility indexes in the umbilical artery (UA-PI). A significant correlation has been found between a reduced head circumference (HC) and the presence of brain injury at MRI (*p* = 0.003). **Conclusions:** Congenital left- and right-sided cardiac obstructive lesions are responsible for fetal hemodynamic changes and brain growth impairment. The correct evaluation of the central nervous system (CNS) in fetuses affected by CHD could be essential as prenatal screening and the prediction of postnatal abnormalities.

## 1. Introduction

Congenital heart diseases (CHDs) represent some of the most frequent fetal and neonatal abnormalities, which seem to affect 9 per 1000 live births [1]. These numbers may underestimate the real prevalence, which includes 20% of the spontaneous miscarriages and 10% of intrauterine demises [2]. CHD involves a huge variety of cardiovascular defects which could have a detrimental effect on neonatal and infant outcomes as well as a great impact on personal and family’s quality of life [3]. An extensive body of evidence has already assessed an association between CHD and neurocognitive impairment, as a direct consequence of the postnatal cardiac surgery on brain development [4,5,6]. Interestingly, the use of neuroimaging techniques, such as magnetic resonance imaging (MRI) and functional-MRI (f-MRI), suggest the hypothesis of brain abnormalities even before birth [7,8,9,10,11,12,13,14].

Therefore, several authors have studied the hemodynamic changes in fetuses affected by various subtypes of CHD [15,16,17,18,19,20]: in particular, they evaluated the presence of hypoplastic left heart syndrome (HLHS) on the onset of cerebral abnormalities and cardiovascular changes. This CHD consists of an inadequate left cardiac output and a negligible flow into the ascending aorta [21]. This leads to a necessary redistribution of energetic substances, as a consequence of this impaired circulation. [15,16,17,18,19,20]. This inadequate hemodynamic flow can severely compromise myelinization and growth of brain cells as well as its microstructure, leading to the risk of a white matter impairment. [12,22]

Consequently, exploring the hemodynamic adaptation of CHD and the possible relationship between heart disease and neurological impairment becomes of paramount importance.

Therefore, the primary aim of this study is to detect potential correlations between the type of CHD and changes in Doppler velocimetry. The secondary aim is to investigate the presence of central nervous system (CNS) abnormalities in fetuses affected by complex CHD.

## 2. Materials and Methods

From July 2010 to July 2020, a cross-sectional study was carried out, recruiting pregnant women referred for fetal echocardiography. The inclusion criteria required a diagnosis of one of the following complex CHD:

1. LSOL (left-sided obstructive lesions): HLHS, aortic stenosis, aortic arch hypoplasia or coarctation.
2. RSOL (right-sided obstructive lesions): pulmonary atresia, tetralogy of Fallot, Ebstein’s anomaly, Tricuspid atresia, pulmonary stenosis.
3. MTC (mixed type of CHD): double outlet right ventricle without pulmonary stenosis, single ventricle, truncus arteriosus, transposition of great arteries.
4. Others (e.g., cardiomyopathy, tumors).

Exclusion criteria included (1) gestational age less than 20 weeks or greater than 40 weeks, (2) cardiac lesion other than the ones listed in the inclusion criteria, (3) age less than 18 years (4) persistent non-sinus rhythm, (5) fetal anemia (6) maternal condition that might affect fetal hemodynamics, such as fetal growth restriction, gestational diabetes, thyroid disease, or pre-eclampsia, (7) presence of any kind of extracardiac anomalies or neurologic malformations detectable with US, MRI and invasive procedures such as amniocentesis, (8) monochorionic twins and (9) chromosomal and sub-chromosomal anomalies, analyzing amniotic fluid samples.

All patients included in the study group as part of a research protocol underwent:

1. Fetal echocardiography;
2. Ultrasound evaluation;
3. MRI of fetal brain;
4. Genetic counseling;
5. Amniocentesis.

### 2.1. Fetal Echocardiography

Fetal echocardiography was conducted according to the International Society of Ultrasound in Obstetrics and Gynecology guideline [23] using a WS80A Elite scanner (Samsung Electronics, Seoul, South Korea) equipped with a 6 MHz curvilinear transducer.

Second-level echocardiography was performed in all fetuses following the sequential and systematic approach of heart evaluation. Multiple two-dimensional views were obtained to evaluate fetal heart anatomy. Doppler flow was employed to evaluate valve competence, stenosis, and shunting. M-mode was used to assess the cardiac rhythm. Doppler color flow mapping was used to identify the umbilical vessels; subsequently, a reduced color scale was used to identify the circle of Willis and the middle cerebral artery (MCA). All the CHD prenatally diagnosed were confirmed with an echocardiography performed postnatally.

### 2.2. Ultrasound Evaluation

All ultrasound evaluations were performed with a Voluson 730 Expert GE or Samsung Elite WS80A machine. Two full-time certified sonographers (F.V., A.G.) performed all the ultrasound scans. A first-trimester evaluation assessed the exact gestational age using the last menstrual period or fetal crown–rump length (CRL) [24].

The following fetal biometric parameters were analyzed: biparietal diameter (BPD), head circumference (HC), abdominal circumference (AC) and femoral length (FL). Estimated fetal weight (EFW) was calculated according to the method of Hadlock et al. [24,25]; both estimated fetal weight and birth weight centile were obtained using local reference curves [26].

IUGR was defined as birth weight (BW) below the 10th percentile for gestational age or estimated fetal weight or abdominal circumference below the 5th percentile at the mid-trimester anomaly scan, in presence of maternal and/or fetal Doppler anomalies [27]. Conversely, a fetus was detected as small for gestational age if the EFW or AC were below the 10th percentile, according to gestational age, or if the Z-score was below 2 [28].

Pulsed-wave Doppler was used to determine blood flow velocities in the umbilical artery (UA) and MCA. The peak systolic velocity, peak diastolic velocity and mean velocity were measured from stable signals during fetal apnea. The pulsatility index (PI) is a measure of vascular resistance in the circulatory bed downstream from the point of Doppler sampling. It is calculated according to the relationship: PI = (systolic velocity − diastolic velocity)/mean velocity. The MCA-PI to UA-PI ratio was labeled as the cerebroplacental (CPR) ratio [29,30].

### 2.3. Magnetic Resonance Imaging

MRI examinations were acquired using a 1.5 T Magnet (Siemens Magnetom Avanto, Erlangen Germany) without maternal–fetal sedation with one or two surface coil phased arrays.

The study protocol included the following sequences with the multiplanar acquisition (axial, coronal, sagittal) [31]:

- T2-weighted HASTE: repetition time (TR) 1500 ms, echo time (TE) 151 ms; slices of 3 mm; FOV 260 × 350 mm; 256 × 256 matrices; time of acquisition (TA) 20 s.
- T1-weighted FLASH 2D: TR 362 ms; TE 4.8 ms; slices 5.5 mm; flip angle 70°; FOV 350 × 300 mm; 256 × 192 matrices; TA 25 to 30 s with and [29] without fat saturation.
- Diffusion weighted imaging: TR 8000 ms; TE 90 ms; inversion time 185 ms; slices of 5 mm; FOV 420 × 300 mm; 192 × 192 matrix; TA 45 s; 3 b-factor per floor: 0.200 and 700 mm^2^/s.

The following parameters were evaluated: biometry (Fronto-Occipital Diameter, cerebral biparietal diameter (BPD), Transverse Cerebellar Diameter, height of the vermis, Antero-Posterior Diameter of the vermis and length of Corpus callosum), ventriculomegaly (VM), gyration, and signal intensity.

### 2.4. Genetic Counseling

Genetic counseling was proposed to all the couples in presence of CHD. Amniocentesis for karyotype and CGH-array was proposed in all CHD cases; fetuses showing chromosomal and copy number variations (CNV) were excluded from the conducted analysis.

### 2.5. Statistical Analysis

The statistical analysis was performed using the Statistical Product and Service Solutions software (SPSS) version 20 for Windows (SPSS Inc., Chicago, IL, USA). Descriptive analyses were presented as frequency with percentage, mean and standard deviation for all variables considered.

We converted the PI measurements into Z-scores using published normative data from a cohort of 72,387 healthy fetuses from the Fetal Medicine Foundation database (https://fetalmedicine.org, accessed on 21 March 2022) [20,32]. In this way, the conducted analyses turn out to be independent of the gestational age. A Z-score equal to 0 refers to the mean of the normal data and a Z-score equal to ±1 and ±2 is at 1 and 2 SDs from the mean, respectively.

Doppler indices were compared between diagnostic groups using one-way ANOVA to determine differences between two groups. Chi-square test and *t*-test were used for intergroup correlations. *p*-values < 0.05 were considered statistically significant.

### 2.6. Ethical Approval

The study was approved by the Institutional Review Board of the Department of Maternal and Child Health and Uro-gynecological Sciences, Sapienza, University of Rome, Policlinico Umberto I, Italy (Report No.: 45/2010) as a quality improvement study with anonymized data. All the patients provided a written informed consent form, and all the followed procedures were in line with the Helsinki declaration’s principles of 1975, as revised in 2000.

## 3. Results

During the study period, 170 individual fetuses suspected of CHD were evaluated by echocardiography. In 143 fetuses, CHD was confirmed. Eighty fetuses were excluded for: gestational age less than 20 weeks (*n* = 3), extracardiac anomalies (*n* = 29), chromosomal anomalies (*n* = 33), non-sinus rhythm (*n* = 5), maternal condition (*n* = 8), and monochorionic twins (*n* = 1; 2 pairs). In particular 120/143 (83%) underwent amniocentesis and a chromosomal anomaly was found in 27% of cases. Sixty-three fetuses were finally included for the analysis and evaluated between 19 and 38 weeks. The specific CHD diagnoses and the mean gestational ages at the time of the fetal echocardiogram are listed in Table 1.

### 3.1. Cerebroplacental Doppler Data

Doppler values were obtained in 46/63 cases. The mean PI Z-scores for the UA and MCA and the mean CPR ratio Z-scores are shown in Table 2.

We did not observe a significant difference in the UA-PI values (*p* = 0.07), even with regard to the RSOL group, which had a higher UA-PI than the other groups. We found a significant difference in the MCA-PI (*p* = 0.036) values among all groups considered, with the LSOL group having a lower MCA-PI than the other ones. We did not observe statistically significant differences in the CPR values among all groups considered (*p* = 0.4343505). We observed a significant reduction in HC measures, as LSOL was associated with lower values.

### 3.2. Brain Abnormalities

MRI was performed in all fetuses. It was found that 36/63 (57.1%) fetuses had signs of brain abnormalities at MRI and in 27/63 (42.9%) brain MRI was normal. Brain alterations are listed in Table 3. Corpus callosum (CC) abnormalities and ventriculomegaly (VM) were present, respectively, in 16/36 (25.4%) and 13/36 (20.6%) of fetuses. We stratified the analysis according to the single groups of CHD (Table 3): most of the cases of brain abnormalities were detected in the MTC and Others groups.

We found a significant correlation between the reduced HC and the presence of brain alterations at MRI (*p* = 0.003). Conversely, we did not achieve statistical significance evaluating the correlation between the detection of brain anomalies and UA-PI, MCA-PI, and CPR values (Table 4).

## 4. Discussion

This study investigates the relationship between the fetal cerebrovascular hemodynamic changes and the presence of CNS abnormalities in fetuses affected by CHD. We have observed that RSOL and LSOL CHD might cause a considerable change in Doppler velocimetry: in our LSOL group, 6/11 (54.5%) fetuses had an HLHS and the analysis of their Dopplers showed low MCA-PI values (*p* = 0.036). Different studies reported the same trend but without significant difference [20,33]. Kaltman et al. [20] also found that only fetuses with HLHS had a lower PIMCA (*p* = 0.001): this result might be attributed to the severity of obstructive lesions of fetuses with LSOL, which was inversely proportional to the amount of cerebral blood delivery. Interestingly, the use of Z-score index was able to completely remove the affect related to gender and gestational age, providing more comparable results.

We also observed registered a tendency to higher elevated UA-PI in fetuses with RSOL (*p* = 0.027), whereas in a previous study, Kaltman et al. reported a similar finding in their study, despite a significant elevation of UA-PI only in fetuses with severe RSOL. This could be related to the severe obstruction of the outflow tracts which could impair the diastolic blood flow in the UA, elevating UA-PI [20].

Evaluating the impaired hemodynamic flow that affected IUGR fetuses instead of SG, we decided to exclude IUGR fetuses from our analysis, because of the possible bias in Doppler velocimetry’s assessment that might intrinsically affect and compromise its course. [29] Evaluating the CPR values, we did not find any statistically significant difference among the CHD subgroups, except for a tendency of lower CPR values in fetuses with LSOL, compared to the other CHD types. Therefore, this could be explained by the assumption that an obstruction of the left outflow tract might impact downstream pulsatility. In light of the above, the use of CPR as an indication of the brain-sparing effect may be inappropriate in the setting of CHD.

The type of CHD contributes to a blood flow distribution, resulting in devastating effects on neurological development [34,35]. In our study, we have found a significant correlation between reduced HC and the presence of brain alterations at MRI: this is in line with the scientific literature which showed a reduction in frontal brain area of fetuses with CHD with neurodevelopmental delay (NDD), in comparison with normal controls. [15,16,17,36,37,38,39]. The exclusion of IUGR fetuses from our study allows us to demonstrate that, in fetuses with CHD, the reduced HC is mainly due to hemodynamic alterations. The etiology of the neurological delay is likely to be complex and multifactorial: some attributed it as a complication of surgery; conversely, it has been already well-established that pre- and peri-operative risk factors account for roughly 30% of poor neuro-developmental outcomes [40,41]. A systematic review examined the prevalence of prenatal brain abnormalities in fetuses with CHD [42]: three studies reported a 28% rate of structural brain abnormalities in fetuses with CHD, including abnormalities in brain’s structure, volume and blood flow. In our study we did not find any statistically significant correlation between the CNS morphological alterations and the type of CHD. The lack of statistically significant data could be related to the limited sample size of LSOL and RSOL subgroups.

Our statistical data analysis showed that up to 57% of fetuses with CHD had brain abnormalities; particularly in the groups of LSOL (63%) and Others (70%). The most frequent alteration was observed in the corpus callosum size, usually related to fetal biometry. This finding was also reported by Ng et al. who carried out a study using tensor-based morphometry: they found a high rate of brain’s volume alteration in infants with CHD compared to healthy control infants. They detected different development in gray, white matter and corpus callosum size, without establishing a direct correlation between the CHD subtype and the morphologic abnormalities. It seems that the regional brain involvement could be related to different oxygen demand and cerebral hemodynamic changes that affected CHD samples [43].

The international guidelines allow the MRI brain evaluation only in fetuses affected by HLHS, despite the consolidated literature evidence of brain abnormalities present in more than 1/3 of CHD fetuses [44,45].

This research represents one of the few studies in the literature which investigate the hemodynamical changes and brain abnormalities in fetuses with complex CHD using two different imaging techniques: US and MRI. Our results confirm the advantages of performing fetal brain MRI in fetuses with complex CHD to characterize and manage the structural and hemodynamic brain modifications even before birth.

Conversely, there are few limitations: first, the relatively small number of cases for the single complex CHD categories, which has prevented a detailed analysis by a single type of CHD. Second, we did not include a control group and specific gestation windows to compare the Doppler velocimetry indexes; this limitation is partially overcome with the use of Z-scores, which allows us to view our data in the context of previously published normal values. The mean MCA-PI Z-scores for the LSOL and RSOL groups were −0.75 and 0.89, respectively. While these values are within the range of normal (within a Z-score range of ±2), they suggest a deviation from the mean of the normal population. In addition, outcome data, such as HC, birth weight, and the MRI scans of the brain at birth, were not obtained due to our inability to follow the antenatal and postnatal progress of these fetuses.

## 5. Conclusions

Our data seem to support that complex CHD impairs the growth of the central nervous system. Left and right-sided cardiac obstructive lesions modify the fetal cerebrovascular resistance. We demonstrated that MCA-PI is lower in fetuses with LSOL, and, according to previous study, we registered higher UA-PI in fetuses with RSOL even if not statistically significant, due to the smaller sample of RSOL group than the other groups. Furthermore, we did not find any statistically significant correlation between the CNS morphological alterations and the type of CHD, but we underline the importance of the study of the fetal brain when a cardiac abnormality is present. Alterations in cerebrovascular blood flow distribution may be associated with the postnatal, neurological abnormalities found in some newborns with complex CHD. Results of the multicenter international children NEUROHEART ongoing study will compare and describe preoperative markers on CHD-affected fetuses in prenatal as well as postnatal brain functional monitoring.

## Figures and Tables

**Table 1:** Descriptive analysis of main patients’ characteristics using mean and standard deviation (SD) or *n* (%).

Main Sample’s Characteristics	Mean ± SD
**Age** (years)	33.6 (5.3)
**Gestational age at evaluation** (weeks)	31.7 (5.4)
**Type of CHD**	***n* (%)**
LSOL	11 (17.5)
RSOL	6 (9.5)
MTC	26 (41.3)
Others	20 (31.7)

LSOL: left-sided obstructive lesions; RSOL: right-sided obstructive lesions; MTC: mixed type of CHD.

**Table 2:** One-way ANOVA Kruskal–Wallis Test using the z-scores of the variables analyzed, according to different types of CHD. CHD: congenital heart disease, HC: head circumference; UA-PI: pulsatility index of umbilical artery; MCA-PI: pulsatility index of middle cerebral artery; CPR: cerebroplacental ratio; LSOL: left-sided obstructive lesions; RSOL: right-sided obstructive lesions; MTC: mixed type of CHD.

US Variables	LSOL Median (IQRSD)	RSOL Median (IQR) m SD	MTC Median (IQR) m SD	OTHERS Median (IQR) m SD	*p*-Value
**UA-PI**	0.38 (2.29)	0.87 (1.88)	−0.28 (2.09)	0.15 (1.50)	0.4076
**MCA-PI**	−0.78 (1.88)	0.93 (1.68)	−0.33 (1.20)	0.34 (1.24)	**0.036**
**CPR**	−1.28 (1.66)	−0.26 (1.42)	−0.43 (1.73)	−0.08 (1.32)	0.4343
**HC**	−1.36 (0.89)	−0.4 (0.80)	−0.81 (0.45)	−0.96 (1.00)	**0.0182**

**Table 3:** Frequencies of brain abnormalities according to the different types of CHD, using number (*n*) and percentage (%). CHD: congenital heart disease; LSOL: left-sided obstructive lesions; RSOL: right-sided obstructive lesions; MTC: mixed type of CHD.

Fetal Brain Abnormalities	LSOL *n* (%)	RSOL *n* (%)	MTC *n* (%)	OTHERS *n* (%)	TOTAL *n* (%)
**Supratentorial diameter**	0	1 (11.1)	5 (55.6)	3 (33.3)	9 (14.3)
**Subtentorial diameter**	3 (37.5)	1 (12.5)	4 (50)	0	8 (12.7)
**Corpus callosum**	5 (31.3)	0	6 (37.5)	5 (31.2)	16 (25.4)
**Subarachnoid spaces**	0	1 (12.5)	4 (50)	3 (37.5)	8 (12.7)
**Gyrification abnormalities**	2 (22.2)	3 (33.4)	4 (44.4)	0	9 (14.3)
**Ventriculomegaly**	3 (23)	0	6 (46.2)	4 (30.8)	13 (20.6)

**Table 4:** Correlations among US descriptors and the presence of brain injury at MRI, using mean (m) and standard deviation (SD). US: ultrasound; MRI: magnetic resonance imaging; CNS: central nervous system; HC: head circumference; UA-PI: pulsatility index of umbilical artery; MCA-PI: pulsatility index of middle cerebral artery; CPR: cerebroplacental ratio.

US Descriptors	No CNS Abnormalities m (SD)	CNS Abnormalities m (SD)	*p*-Value
**HC**	307.8 (44)	264 (49.5)	**0.003**
**UA-PI**	0.9 (0.18)	1.07 (0.24)	0.15
**MCA-PI**	1.78 (0.32)	1.72 (0.33)	0.59
**CPR**	1.88 (0.44)	1.67 (0.47)	0.13

## Data Availability

Not applicable.
